# Enhancing Retinal Resilience: The Neuroprotective Promise of BDNF in Diabetic Retinopathy

**DOI:** 10.3390/life15020263

**Published:** 2025-02-09

**Authors:** Daniela Maria Tanase, Emilia Valasciuc, Evelina Maria Gosav, Mariana Floria, Oana Nicoleta Buliga-Finis, Anca Ouatu, Andrei Ionut Cucu, Tina Botoc, Claudia Florida Costea

**Affiliations:** 1 Department of Internal Medicine, “Grigore T. Popa” University of Medicine and Pharmacy, 700115 Iasi, Romania; daniela.tanase@umfiasi.ro (D.M.T.); dr.emiliavalasciuc@gmail.com (E.V.); dr.evelinagosav@gmail.com (E.M.G.); oana_finish@yahoo.com (O.N.B.-F.); ank_mihailescu@yahoo.com (A.O.); 2 Internal Medicine Clinic, “St. Spiridon” County Clinical Emergency Hospital, 700111 Iasi, Romania; 3 Department of Biomedical Sciences, Faculty of Medicine and Biological Sciences, “Ștefan cel Mare” University, 720229 Suceava, Romania; andrei.cucu@usm.ro; 4 Department of Neurosurgery, “Prof. Dr. Nicolae Oblu” Emergency Clinical Hospital, 700309 Iasi, Romania; 5 Department of Ophthalmology, “Grigore T. Popa” University of Medicine and Pharmacy, 700115 Iasi, Romania; tina.botoc@gmail.com (T.B.); costea10@yahoo.com (C.F.C.); 6 2nd Ophthalmology Clinic, “Prof. Dr. Nicolae Oblu” Emergency Clinical Hospital, 700309 Iasi, Romania

**Keywords:** diabetes mellitus, T2DM, diabetic retinopathy, brain-derived neurotrophic factor, BDNF

## Abstract

Diabetic retinopathy (DR), a leading cause of vision impairment worldwide, is characterized by progressive damage to the retina due to prolonged hyperglycemia. Despite advances in treatment, current interventions largely target late-stage vascular complications, leaving underlying neurodegenerative processes insufficiently addressed. This article explores the crucial role in neuronal survival, axonal growth, and synaptic plasticity and the neuroprotective potential of Brain-Derived Neurotrophic Factor (BDNF) as a therapeutic strategy for enhancing retinal resilience in DR. Furthermore, it discusses innovative delivery methods for BDNF, such as gene therapy and nanocarriers, which may overcome the challenges of achieving sustained and targeted therapeutic levels in the retina, focusing on early intervention to preserve retinal function and prevent vision loss.

## 1. Introduction

Diabetic retinopathy (DR) is a major microvascular complication of diabetes and the leading cause of visual impairment and blindness worldwide [1]. Its prevalence continues to increase in the working class population, leading to severe healthcare expenses and socioeconomic burden globally [2].

As many pathophysiological mechanisms are involved in its development, DR includes inflammatory, neurological, and microvascular changes [3]. Combining the clinical manifestations and pathophysiological aspects, DR can be classified as proliferative diabetic retinopathy (PDR) and non-proliferative diabetic retinopathy (NPDR) [4]. NPDR is usually asymptomatic and includes blood vessel microaneurysms, retinal hemorrhages, intraretinal microvascular abnormalities (IRMA), venous caliber changes, which may lead to hypoperfusion, and ischemia [5], while PDR involves abnormal activity of angiogenic factors, leading to neovascularization [4]. The fragility of the newly formed blood vessels causes bleeding on the retinal surface or in the vitreous body, together with macular edema (ME), which is a continual fluid accumulation that affects the center of the macula, causing permanent visual impairment [6].

Current treatments for DR, such as anti-vascular endothelial growth factor (anti-VEGF) therapies and laser photocoagulation, primarily target late-stage vascular complications [7]. However, these interventions do not address the underlying neuronal damage and often fail to prevent disease progression [7]. This highlights the importance of exploring alternative therapeutic approaches that can preserve retinal function and integrity earlier in the disease course.

Brain-derived neurotrophic factor (BDNF), a key neurotrophin involved in neuronal survival, plasticity, and repair, has emerged as a promising candidate in this context [8,9]. BDNF plays a pivotal role in maintaining retinal health by promoting the survival of retinal ganglion cells and modulating neurovascular interactions [8,9]. Evidence suggests that alterations in BDNF levels contribute to the neurodegenerative processes observed in DR, linking metabolic dysregulation to retinal dysfunction [8,9].

This article explores the neuroprotective potential of BDNF in the management of diabetic retinopathy, emphasizing its role in mitigating neuronal and vascular damage, aiming to uncover novel pathways for preserving vision and addressing the complex pathophysiology of DR.

## 2. Structure and Expression of BDNF

BDNF secretion, receptor interactions, and signaling pathways constitute a finely tuned system critical for neuronal survival, differentiation, and synaptic plasticity [8,9]. Activity-dependent secretion ensures the spatial and temporal precision of BDNF effects, while receptor-specific signaling pathways enable diverse biological outcomes [8,9]. The interplay between tropomyosin receptor kinase B (TrkB) and p75 neurotrophin receptor (p75NTR) signaling reflects a dynamic balance between growth-promoting and inhibitory effects, which is essential for maintaining neural homeostasis [8,9]. Disruptions in this system are linked to a wide range of neurological and psychiatric conditions, highlighting the therapeutic potential of modulating BDNF signaling to restore neural function and resilience [8,9].

### 2.1. Origin, Structure and Synthesis

BDNF is a constituent of the neurotrophin family of growth factors, which includes nerve growth factor (NGF), neurotrophin-3 (NT-3), and neurotrophin-4/5 (NT-4/5) [8]. Like other neurotrophins, BDNF is synthesized in the endoplasmic reticulum (ER) as a glycosylated precursor protein called proBDNF that is transported through the Golgi apparatus where it is sorted into vesicles by lipid raft-associated receptor carboxypeptidase E (CPE) [10]. Following its post-synaptic regulation, proBDNF is released and subsequently undergoes enzymatic cleavage by protein convertases, resulting in its active mature form, mBDNF [11,12].

From a structural perspective, BDNF exhibits roughly half of the amino acid similarity with NGF, NT-3, and NT-4/5 [8]. All neurotrophins are found as non-covalently associated homodimers, characterized by the presence of a signal peptide and a pro-region containing glycosylation sites [8]. In rats, the BDNF gene is situated on chromosome 11 and is regulated by various tissue-specific promoters along with transcription factors like cyclic adenosine monophosphate (cAMP) response-element binding protein (CREB) and calcium-responsive transcription factor (CaRF) [13,14]. The expression of the BDNF gene is activated through mechanisms induced by neuronal activity, showcasing different exons that express specifically in various tissues [9,15]. In humans, the BDNF structure is similar to that described in rats, and the gene located at chromosome 11p14.1 comprises 11 exons and nine distinct promoters, with the principal coding sequence for the precursor protein residing in exon IX [9,15].

The transcription of BDNF is meticulously controlled at multiple levels by neuronal activity, particularly through calcium influx [9,10]. The secretion of BDNF is tightly controlled at multiple levels, including transcription, translation, storage, and release. BDNF is synthesized as a precursor protein, pro-BDNF, which undergoes intracellular processing and can also be secreted in its unprocessed form [10].

This process results in the generation of multiple spliced mRNA isoforms capable of being transported to dendrites [12]. Within active neuronal regions, BDNF can undergo local translation and secretion, thereby facilitating synaptic plasticity and the formation of both developing and established neural circuits [12]. During developmental stages, BDNF levels are minimal in the fetal brain but rise significantly after birth before experiencing a decline in adulthood [9,16].

BDNF is essential for functioning within both the central nervous system (CNS) and peripheral nervous system (PNS), gaining recognition for its neuroprotective properties [9]. For BDNF to fulfill its biological roles, it must be secreted by the appropriate cells at designated times and locations [9]. Following its synthesis, BDNF is stored in dense-core vesicles (DCVs) and is released when there is an increase in cytoplasmic calcium (Ca^2+^) levels [9]. This release occurs through two mechanisms: the influx of extracellular Ca^2+^ or the release of Ca^2+^ from intracellular organelles. In brain cells or neuronal cultures, glutamate-induced BDNF release depends on the mobilization of Ca^2+^ from internal reserves, whereas the secretion of BDNF initiated by action potentials relies on external sources of Ca^2+^ [9,11].

### 2.2. Regulated Secretion: Receptors and Signaling Cascades

Upon release into circulation, BDNF binds preferentially to TrkB, which is a transmembrane receptor endowed with tyrosine kinase activity that undergoes dimerization and autophosphorylation at specific tyrosine residues within its intracellular domain, triggering various downstream signaling pathways including the mitogen-activated protein kinase/extracellular signal-regulated kinase (MAPK/ERK) pathway, the phosphoinositide 3-kinase (PI3K)/Akt pathway, and the phospholipase C-gamma- protein kinase C (PLC-γ-PKC) pathway, which play critical roles in neuronal growth and differentiation, synaptic plasticity, and cognitive processes such as learning and memory [9,11,17,18].

The MAPK/ERK pathway is critical for promoting neuronal survival and differentiation. Upon TrkB activation, adaptor proteins recruit SOS, a guanine nucleotide exchange factor, leading to the activation of Ras [18,19]. This activation sets off a kinase cascade that includes RAF, MEK, and ERK, ultimately facilitating transcriptional programs linked to cell growth and survival [18,19].

The PI3K/Akt pathway is essential for neuronal survival and metabolic regulation. PI3K activation leads to the generation of phosphatidylinositol-3,4,5-triphosphate (PIP3), which subsequently recruits and activates Akt [20,21]. Once activated, Akt phosphorylates downstream targets like mTOR to stimulate protein synthesis, prevent apoptosis, and support cell viability [20,21].

Additionally, the PLC-γ pathway, meanwhile, facilitates synaptic plasticity by increasing intracellular calcium levels and activating PKC [9,18,22]. TrkB-mediated PLC-γ activation leads to the hydrolysis of phosphatidylinositol-4,5-bisphosphate (PIP2) into inositol-1,4,5-trisphosphate (IP3) and diacylglycerol (DAG), with IP3 stimulating calcium release from the ER and DAG activating PKC [9,18,22]. These signaling events enhance long-term potentiation (LTP), a cellular mechanism underlying learning and memory [9,18,22].

**Figure 1:**
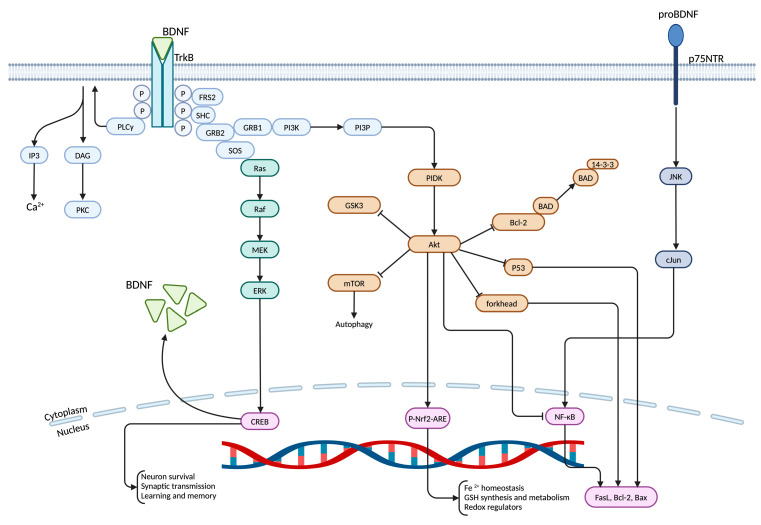
The signaling cascade of BDNF. CREB (cyclic adenosine monophosphate (cAMP) response-element binding protein); PI3K (phosphoinositide 3-kinase); JNK (Jun kinase); AGEs (advanced glycation end products); Nrf2 (nuclear factor erythroid 2–related factor 2).

Conversely, proBDNF interacts with the p75^NTR^, which belongs to the tumor necrosis factor receptor (TNFR) superfamily [23,24]. This receptor features a distinctive extracellular domain that aids in neurotrophin binding alongside a cytoplasmic domain capable of activating signaling pathways such as Jun kinase (JNK) and nuclear factor kappa-light-chain-enhancer of activated B cells (NF-κB), which promote apoptosis or inflammatory responses [23,24]. Notably, signaling through p75^NTR^ can yield contrasting effects promoting neuronal survival during development while potentially inducing cell death under pathological circumstances, this balance being critical for maintaining neuronal homeostasis, particularly during development and in response to injury [23,24] (Figure 1).

Furthermore, BDNF synthesis extends beyond just neuronal cells and it is also produced by immune cells including T cells, B cells, and monocytes along with expression in organs such as the heart, skeletal muscles, liver, spleen, and thymus [11,15]. The regulation of BDNF expression occurs developmentally and is subject to influence from environmental stimuli such as physical exercise, hypoxia conditions, stressors, and seizure activities, which can enhance its production [11,15].

Although neurons are the primary cells documented in relation to BDNF release, research indicates that astrocytes and microglia also possess the ability to secrete this neurotrophic factor [11]. The dynamics of BDNF secretion are modulated by a variety of stimuli, including depolarization induced by heightened extracellular potassium (K⁺) concentrations, high-frequency stimulation (HFS), and theta-burst stimulation (TBS) [11]. Furthermore, other factors, such as neuropeptides (e.g., neurotrophins, CGRP) and compounds like glutamate, ATP, capsaicin, and adenosine, can also stimulate the release of BDNF [11].

### 2.3. Biological Neuroprotective and Neuronal Differentiation Functions

BDNF is a member of a family of growth factors, called neurotrophins [25], involved in regulating the survival, growth, and maintenance of neurons and also in the promotion of angiogenesis and fibrosis [26]. Expressed at its highest level in the brain of humans [10], it induces LTP, which is essential for learning and memory [26]. In the retina, BDNF is produced by retinal ganglion cells (RGCs), amacrine cells, astrocytes, Müller cells, and photoreceptors [27].

BDNF has multiple roles such as protecting the retina cells from injuries caused by hypoxia and glucose deprivation [28], the long-term protection of RGCs, antiapoptosis, and maintaining visual function in ocular hypertension [29]. BDNF’s receptors include TrkB, expressed on RGCs, and p75^NTR^ [30], which is expressed preferentially on glial cells, as RGCs express TrkB but do not express p75^NTR^ [31]. The administration of mouse monoclonal antibodies, which act as TrkB exogenous selective agonists, induces antiapoptotic activity, reinforcing BDNF’s multipurpose role [32,33]. As BDNF is known to play an important role in neuronal survival [34], it has been shown that the administration of exogenous BDNF following optic nerve injury in rodents with induced glaucoma and in glaucoma patients slows RGC degeneration, glaucoma being characterized by RGCs and optic nerve atrophy [29]. The administration of various neurotrophic factors, including BDNF, exerts neuroprotective effects in animal models of diabetes and also prevents preexisting retinal dysfunctions from worsening in diabetic patients [23].

BDNF plays an important role in increasing the number of neurons and their survival rate [35]. One of the mechanisms that make it possible would be the mediation of BDNF effects via gamma-aminobutyric acid (GABA)-ergic transmission. BDNF promotes the inhibitory networks of GABA transmission upon binding to TrkB receptors with a beneficial effect on the augmentation of progenitor cells for neuronal differentiation [25].

Another hypothesis regarding this context would be the BDNF-induced activation of Ca^2+^/calmodulin-dependent protein kinase II (CaMKII) in BDNF-mediated responses and the p38 subfamily of MAPK activation [36,37,38]. It is speculated that MAPK-activated protein kinase 2 (MAPKAPK-2) is possibly the kinase activated during this pathway and that it phosphorylates the key positive regulatory site of the CREB protein, leading to its activation. CREB is the prime mediator of BDNF responses, including synaptic plasticity and neuronal survival [36,37,38].

BDNF plays a pivotal role in the development of synaptic connections and neuronal circuits through intracellular cAMP signaling. Together with other cAMP-dependent pathways, BDNF plays important roles in neuronal growth and development [15,39].

## 3. Clinical Implications of BDNF in Diabetic Retinopathy

According to some studies [40,41] BDNF can be used as a diagnostic marker of DR in its early stages [31], as its level in serum and in aqueous humor is strongly decreased even before the clinical manifestations of DR [42]. Also, the plasmatic concentrations can be used as an indicator of DR development [43], a correlation being proven between serum BDNF concentrations and the duration and evolution of the disease [44]. This way, the serum BDNF levels of patients with PDR are lower than those of patients with non-PDR and lower in patients without DR [42].

### 3.1. Diabetic Retinopathy Development

Hyperglycemia is the main cause of tissue damage [45], activating multiple metabolic pathways, such as the polyol pathway, the PKC pathway, and the hexosamine pathway, and also leads to advanced glycation end-product (AGE) accumulation [46].

A high glucose level causes non-enzymatic glycosylation, which leads to AGE formation, which contributes to oxidative-stress-induced damage to retinal cells secondary to intracellular reactive oxygen species (ROS) augmentation [10]. In addition, the alteration of pigment epithelium-derived factor (PEDF) occurs, resulting in inflammation and microvascular damage [47]. AGE formation also activates the enzyme complex nicotinamide adenine dinucleotide phosphate (NADPH) oxidase and NF-kB, promoting further inflammatory mediated cell damage [10,47]. AGE precursors, once released in the extracellular compartment, cause circulating protein alterations, which later, by binding to AGE receptors, activate them, leading to proinflammatory cytokines and growth factor accumulation (vascular endothelial growth factor-VEGF), angiopoietins, tumor necrosis factor (TNF), interleukins (ILs), and matrix metalloproteinases (MMPs) and microvascular changes [48,49].

The metabolic polyol pathway consists of sorbitol production from glucose, with NADPH consumption [50]. Then, sorbitol is metabolized to fructose by sorbitol dehydrogenase using the cofactor nicotinamide adenine dinucleotide (NAD^+^) under the regulation of the aldose reductase (AR) enzyme [47]. High levels of sorbitol become damaging to retinal cells by causing an intracellular osmotic imbalance. Also, the metabolism of fructose produces 3-deoxyglucosone (3DG) and fructose-3-phosphate (F3P), which subsequently escalates oxidative stress (OS) in retinal cells [47].

The third mechanism involved in DR resides in the PKC pathway. In this pathway, under the constant hyperglycemia, the synthesis of diacylglycerol (the endogenous activator of PKC) is elevated and leads to the activation of the isoforms of protein kinase-C, -β, -δ, and -α and p38 mitogen-activated protein kinase, causing important structural and functional abnormalities [48,51]. The activation of PKC implies NADPH oxidase activation, after which the redox reaction starts with both ROS and AGEs accumulation in various vascular cells, including pericytes, endothelial cells, smooth muscle cells, and podocytes, leading to cell death and apoptosis [52]. Generated vascular dysfunction promotes diabetic microvascular complications, such as blood flow alterations, extracellular matrix synthesis and basement membrane thickening [53,54], increased endothelial permeability [55], and angiogenic growth factors synthesis [56].

**Figure 2:**
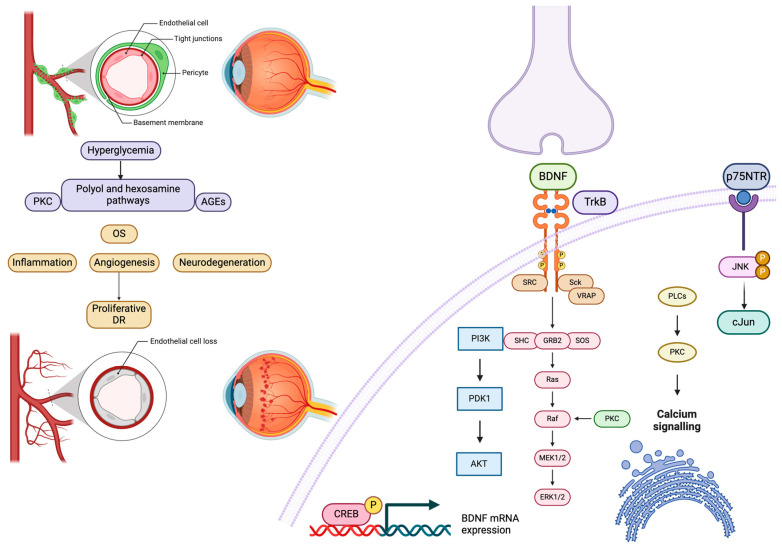
The pathophysiology of diabetic retinopathy (DR) highlights the neuroprotective role of BDNF in mitigating its effects on promoting cell survival, neuronal plasticity and repair, and calcium signaling. CREB (cyclic adenosine monophosphate (cAMP) response-element binding protein); PI3K (phosphoinositide 3-kinase); JNK (Jun kinase); AGEs (advanced glycation end products).

The last mentioned mechanism is the hexosamine pathway, involved in the conversion of glucose to uridine diphosphate N-acetyl glucosamine (UDP-Glc-NAc), a key substrate for the glycosylation of proteins and lipids [57,58]. Glucose uptake and entry into the pathway is followed by phosphorylation to glucose-6 phosphate (G6P), which is further isomerized to fructose-6-phosphate (F6P) by phosphoglucose isomerase. F6P is then converted to glucosamine-6-phosphate (GlcN-6P) by glutamine-fructose-6-phosphate aminotransferase (GFAT), and finally, after acetylation and isomerization, to UDP-Glc-NAc [57,58]. High glucosamine generated by the activation of the hexosamine pathway stimulates the overproduction of ROS at the mitochondrial level, causing OS, elevated vascular permeability, and angiogenesis [51] (Figure 2).

The overabundance of superoxide in the mitochondria sustaining OS links all these metabolic pathways [47]. In addition, hyperglycemia-mediated mitochondrial dysfunction is also believed to be correlated with the overproduction of ROS in DR [51]. OS plays a crucial role in the development of DR due to the high vascularization of the retina and chronic hyperglycemia, which makes the microvessels even more susceptible to OS [59]. The disruption of redox homeostasis leads to optic nerve degeneration and neuronal death in the retina by reducing mitochondrial energy production and byproduct accumulation [60,61]. Consequently, OS induces basal membrane thickening, pericyte apoptosis, and mitochondrial dysfunction, collectively contributing to blood–retina membrane (BRB) impairment and progression to advanced DR [62]. This is followed by retinal ischemia and VEGF upregulation through the activation of hypoxia-inducible factor 1 (HIF-1) [63,64,65]. VEGF, alongside angiopoietins (Ang-1, Ang-2), are believed to increase vascular permeability and to promote endothelial cell proliferation secondary to its angiogenic properties [46] and play a major role in the progression of PDR and Diabetic Macular Edema (DME), the major cause of vision impairment in diabetic patients and an abnormal accumulation of fluid in the macula under diabetic conditions, leading to increased central retinal/macular thickness [66]. Mitochondrial dysfunction also affects the activity of optic nerve cells, the activity of photoreceptors, and ROS accumulation and leads to their deficient functioning [60,67].

Oxidative stress also leads to abnormal epigenetic modifications in cells [51]. Unlike inherited genetic modifications, which are static throughout the life course, epigenetic changes are dynamic and vary depending on the tissue and the disease [68,69,70]. The major epigenetic modifications with retinal involvement are the methylation of DNA and histone modifications [68,69,70].

The methylation status of DNA is strongly correlated with normal retinal development, maintaining specific gene expression in both photoreceptors and non-photoreceptor cells [71]. However, abnormal DNA methylation is associated with the development of age-related macular degeneration (AMD) [72], DR [73], and retinitis pigmentosa development [74]. In diabetic patients, hyperglycemia, hyperhomocysteinemia, and hyperlipidemia often lead to DNA methyltransferases (Dnmts) and ten-eleven translocation dioxygenases (Tets), resulting in aberrant DNA methylation. This aberrant methylation increases ROS levels and triggers the production of chemokines, cytokines, acute phase proteins, and VEGF, contributing to DR via OS, inflammation, and neovascularization [71]. Additionally, OS during DR can also affect DNA methylation, becoming a major reason for “metabolic memory” development, a condition when diabetes-related complications continue to develop despite normalized blood glucose levels [75].

Histone modifications, a type of post-translational modification (PTM), encompass processes such as methylation, acetylation, ubiquitination, and phosphorylation. These modifications play a significant role in DR development by influencing inflammation, OS, mitochondrial dysfunction, cell apoptosis, and neurodegeneration [69]. PTMs are crucial in regulating genes associated with the pathogenesis of diabetes, with several specific modifications identified in the monocytes and lymphocytes of diabetic patients [76]. These modifications can alter chromatin structure and gene expression, thereby contributing to the complex molecular mechanisms underlying DR and its progression [76].

OS disbalances pancreatic β-cell homeostasis, leading to cellular dysfunction and cellular apoptosis. Even if autophagy has been recognized as a mechanism promoting healthy lifespan and longevity, dysregulated autophagy, as in the diabetic retina, contributes to apoptotic retinal cell death, leading to further damage [77]. There are three types of autophagy: macroautophagy, chaperone-mediated autophagy (CMA), and microautophagy [78].

In macroautophagy, a double-membrane structure called an autophagosome is formed, and the entire process is regulated by the ATG (autophagy-related) protein family. ULK1 kinase forms a complex with ATG13, ATG101, and FIP200 (a mammalian autophagy factor), leading to PI3K complex formation. Next, ATG7 mediates the binding of ATG12, ATG5, ATG16L, and phosphatidylethanolamine to light chain 3 protein (LC3) to form autophagosome binding LC3 (LC3-II) [78]. Further, the autophagosome fuses with the lysosome to generate autophagolysosome or autolysosome, degrading its contents [77].

CMA involves the participation of HSC70 (heat shock-cognate chaperone 70 KDa) and LAMP-2A (lysosomal associated-membrane protein 2A). LAMP-2A recognizes chaperone proteins and promotes the expansion and translocation of proteins in the lysosome [79]. In microautophagy, the lysosome membrane directly absorbs the components in the cytoplasm to achieve the purpose of clearance [78].

### 3.2. Retinal Neurodegeneration

Neuronal pathophysiology also plays an important role in DR evolution. Hyperglycemia-induced downregulation of neurotrophic factors such as NGF, pigment epithelium-derived factor (PEDF), interphotoreceptor retinoid-binding protein (IRBP), and somatostatin may contribute to or even initiate neuronal apoptosis and capillary damage [62].

Retinal neurodegeneration is considered an early event in the pathogenesis of DR, preceding vascular lesions [80]. All retinal layers (ganglion, bipolar, amacrine, and photoreceptor cell), demonstrate altered functions even prior to microangiopathy [3]. Hyperglycemia-induced neurodegeneration consists of reactive gliosis, low neuronal function, and cell apoptosis, and has been observed to occur before microangiopathy development in experimental models of DR and in the retina of diabetic donors [80]. As a consequence, ganglion, amacrine cell, and photoreceptor apoptosis lead to reduced thickness of inner retinal layers and the nerve fiber layer, which can be easily detected by optical coherence tomography (OCT) [80].

Activated microglia also play a key role in DR development and are also involved in the neurodegeneration process [81]. Their dysregulation leads to a shift from pro-survival to pro-neurotoxicity, resulting in transcriptional changes, mediated via the NF-κB and ERK signaling pathways, and the release of neurotoxic factors such as caspases, nitrous oxide, and glutamate [82]. The production of proinflammatory cytokines also plays a role in neurodegeneration in DR [83]. These cytokines lead to the breakdown of endothelial cell–cell junctions, resulting in the activation of microglia, followed by the increased apoptosis of ganglion cells and amacrine cells, which disrupts synaptic integrity and contributes to synaptic degeneration [48].

Chronic inflammation plays a central role in the pathogenesis of diabetic retinopathy (DR) by disrupting the BRB, promoting vascular damage, and driving neurodegeneration and apoptosis (Table 1) [84,85].

Persistent hyperglycemia triggers the release of pro-inflammatory cytokines such as TNF-α, IL-1β, and IL-6, which increase OS and endothelial dysfunction, clinically manifested through increased vascular permeability [84]. This leads to leukostasis, where activated leukocytes adhere to the retinal vasculature, further compromising blood flow and contributing to capillary dropout [84,85]. Inflammation also upregulates adhesion molecules like ICAM-1 and VCAM-1, facilitating leukocyte infiltration and sustained immune activation. Additionally, glial cells, including Müller cells and microglia, become overactivated, exacerbating neuroinflammation and retinal damage [84,85]. Increased VEGF levels due to chronic inflammation and hypoxia stimulate pathological neovascularization, worsening disease progression [84]. Targeting inflammatory pathways, such as blocking IL-17A or inhibiting NF-κB, has shown potential in experimental models [84,85]. Understanding and controlling chronic inflammation could lead to more effective therapies for preventing DR progression.

Increasing evidence suggests that both nutrition and inflammation play pivotal roles in the onset and progression of DR, influencing oxidative stress, vascular integrity, and inflammatory responses [85,87]. Understanding the interplay between these factors could lead to more comprehensive treatment approaches beyond conventional therapies. Essential nutrients such as omega-3 fatty acids, vitamin D, and antioxidants play protective roles by reducing inflammation and OS, stabilizing endothelial function, and preserving the BRB [88]. In contrast, diets high in refined sugars and saturated fats contribute to metabolic dysregulation and chronic low-grade inflammation, worsening retinal damage [88].

Recently, a Prognostic Nutritional Index (PNI) has been identified as a crucial potential marker in assessing the risk of microvascular complications, including DR [87]. Research by Aktas et al. indicates that patients with lower PNI levels exhibit a higher prevalence of DR, suggesting that poor nutritional status exacerbates disease severity [87]. This new tool shows promise not only for DR severity assessment but also for other diabetic microvascular complications [87]. Moving forward, personalized nutritional plans alongside anti-inflammatory strategies could serve as a novel therapeutic avenue to prevent and slow DR progression.

### 3.3. Molecular Interplay of BDNF in Diabetic Retinopathy Development

Since DR presents a multifactorial and interdependent pathophysiological process, the specific action of BDNF must be particularized according to the previously described pathophysiological stages.

Regarding its neuroprotective effects, BDNF helps prevent RGC and photoreceptor apoptosis, which are commonly observed in DR [29]. By binding to its high-affinity receptor, TrkB, BDNF triggers downstream intracellular signaling cascades, such as the PI3K/Akt and MAPK/ERK pathways, which prevent apoptosis by inhibiting pro-apoptotic factors such as caspase-3, promoting neuronal survival [9,11,17,18]. Additionally, BDNF enhances the activity of CREB, promoting the expression of anti-apoptotic and neuroprotective genes and enhancing synaptic plasticity, ensuring the preservation of functional neural circuits in the diabetic retina [36,37,38]. In diabetic retinas, the downregulation of BDNF-TrkB signaling has been associated with increased oxidative stress, mitochondrial dysfunction, and inflammation, all of which contribute to retinal neurodegeneration [25]. This is particularly important, as neurodegeneration is an early event in DR progression, occurring before vascular abnormalities [7].

The chronic inflammation environment in DR progression consists of increased levels of pro-inflammatory cytokines such as TNF-α, IL-1β, and IL-6, which disrupt retinal homeostasis by increased OS [10,47]. BDNF has anti-inflammatory effects by reducing inflammatory cytokines and furthermore by suppressing microglial activation, preventing excessive immune responses that could exacerbate neuronal and vascular inflammation-induced injury in the retina [48,86]. The activation of STAT3 by BDNF additionally suppresses pro-inflammatory cytokine production while enhancing neuroprotective gene expression [89]. Furthermore, BDNF modulates the AMPK pathway, which regulates cellular energy metabolism and mitigates OS-induced damage [90]. The ability of BDNF to counteract hyperglycemia-induced damage via these pathways underscores its therapeutic potential in DR [91].

Regarding vascular stability and anti-angiogenic effects, BDNF plays a crucial role in maintaining the BRB integrity by regulating endothelial tight junction proteins such as ZO-1 and occludin, which are especially important in preventing DME, a severe complication of DR [84]. Moreover, by counteracting VEGF-mediated neovascularization, BDNF inhibits abnormal blood vessel formation, reducing the risk of retinal hemorrhages and fibrosis, hallmarks of PDR [84].

As BDNF enhances antioxidant defense mechanisms by increasing the activity of superoxide dismutase (SOD) and other antioxidant enzymes, which help neutralize ROS, it also supports mitochondrial bioenergetics, reducing metabolic stress on retinal neurons and endothelial cells [84]. By maintaining adequate ATP production, BDNF improves retinal cells survival under hyperglycemic conditions.

### 3.4. The Premise of BDNF in Diabetic Retinopathy Management

BDNF has been extensively studied for its potential role in DR, with both clinical and preclinical evidence supporting its neuroprotective effects. Preclinical research has shown that BDNF levels are significantly reduced in the serum, vitreous, and retinal tissue of diabetic patients and animal models, suggesting a key role in DR pathogenesis [40,41,42].

In their studies, both Guo et al. [41] and Liu et al. [40] showed that BDNF levels are significantly lower in diabetic patients without DR compared to a control group and that a low level of serum BDNF can be considered a risk factor for DR development [40]. In their study, Taşlipinar Uzel et al. compared BDNF concentrations in the aqueous humor of diabetic patients without retinopathy and with non-PDR, and these were lower than in the control group values [42].

Animal models have provided valuable insights into the mechanisms by which BDNF exerts its effects in DR, highlighting its neuroprotective and vascular-stabilizing properties. In streptozotocin (STZ)-induced diabetic rodent models, the intravitreal administration of BDNF preserved RGC, reduced oxidative stress, and prevented early-stage neurodegeneration, an effect mediated through TrkB receptor activation and downstream PI3K/Akt and MAPK/ERK signaling pathways [92]. Furthermore, BDNF-treated diabetic mice exhibited improved synaptic plasticity and retinal electrophysiology, demonstrating its functional significance beyond morphological preservation [93].

Despite BDNF’s neuroprotective roles in diabetes, it has been noticed that hyperglycemia induces numerous alterations and the downregulation of BDNF expression [25], also partially mediated by the TrkB levels [94,95]. One of these alterations is linked to the dysfunction of the hypothalamic–pituitary–adrenal (HPA) axis and elevated levels of glucocorticoids in the hyperglycemic state [96]. Numakawa et al. have established the connection between steroid hormone levels and BDNF expression, suggesting that elevated concentrations of glucocorticoids downregulate the expression of BDNF [97,98]. These changes are possible because of glucocorticoid interactions with TrkB receptors, leading to synaptic dysfunction, neuronal apoptosis, and the downregulation of BDNF-induced corticotrophin-releasing hormone (CRH) release [25]. Since CRH is a known insulinotropic hormone, a hyperglycemic state occurs, intensifying the BDNF downregulation as a consequence [99,100]. In addition, elevated levels of glucocorticoids lead to MAPK-ERK pathway inhibition, diminishing the neuroprotective effects mediated by BDNF via this pathway [25]. Also, the inhibition of PLC-gamma pathway activation subsequently leads to decreased glutamate release. This results in alterations in the normal physiology of neuronal development and the regulation of synaptic transmissions, hence causing neuronal dysfunction [25].

However, BDNF’s rapid degradation and limited bioavailability have posed challenges for clinical translation. To overcome these limitations, gene therapy approaches using adeno-associated viral (AAV) vectors have been explored, achieving sustained BDNF expression in retinal cells and prolonged neuroprotection in diabetic animal models [10]. These findings collectively suggest that BDNF-based therapies, particularly in combination with anti-VEGF treatments, could offer a dual approach, targeting both neurodegeneration and vascular dysfunction in DR. Still a promising and developing molecule, further human trials are needed to evaluate the long-term safety, efficacy, and optimal delivery methods for BDNF in clinical settings.

## 4. Treatment

While current treatment modalities, such as anti-VEGF therapy and laser photocoagulation, primarily target late-stage vascular complications, they fail to address the neurodegenerative processes occurring in the retina [7]. Because insufficient amounts of BDNF lead to neuro-retinal apoptosis and degeneration, BDNF-based treatment emerged as a promising therapeutic agent due to its multifaceted neuroprotective and neuroregenerative properties, making it a potentially superior or complementary option for DR treatment when compared to existing therapies.

### 4.1. Pharmacological Approaches

Liu et al. have shown that BDNF neuroprotection in the retina is concentration-dependent, but, in higher doses of BDNF (>100 ng/mL), neuroprotection is not improved, due to a limited number of TrkB receptors [94]. There are a few methods of BDNF administration, such as intravitreal injections, which have shown good short-term results in RGC survival, but because of the short half-life of BDNF protein, this result did not last [101]. The intravitreal administration of BDNF in diabetic rat models also leads to the prevention of dopaminergic amacrine cells from neurodegeneration and lowers the tyrosine hydroxylase (TyrH) activity, improving neuronal survival [102]. Other proposed methods were to dissolve BDNF in 0.5% hyaluronic acid [10] or to merge BDNF to a molecule before administration, such as leucine-rich repeat and immunoglobulin domain-containing protein 1 (LINGO-1) antagonist [103,104]. This combination can provide long-term protection for RGCs. BDNF can also be combined with other growth factors or biological molecules such as N-tert-butyl-(2-sulfophenyl)-nitrone (S-PBN), improving neuronal survival up to 68%, or with NOS-inhibitor N-ω-nitro-L-arginine methyl ester (L-NAME), improving survival up to 55%. Furthermore, the combination of GDNF and BDNF provides more protection for RGCs than either factor alone, as they target different cellular pathways in the retina to enhance RGC survival [101]. Another method of BDNF administration would be gene delivery using a viral vector. Several viruses such as Adenoviruses, Adeno-Associated viruses (AAVs), lentivirus, herpes simplex virus (HSV), Vaccinia Virus, and Sendai viruses are known to be used as gene transfer vectors in the majority of gene therapy clinical trials [10], HSV being considered as a promising carrier because of its ability to infect both quiescent and proliferating cells, including neurons [101]. BDNF gene therapy is another potential method of BDNF delivery, with Müller cells and RGCs as possible targets for gene transduction [10]. The objectives of gene therapy in DR include targeting the angiogenic factors involved in neovascularization and preventing vascular and neuronal degeneration in the early stages of DR. However, clinical trials are still in their early stages of development due to the complex pathogenesis of DR and challenges related to safety, vector delivery specificity, patient selection, and outcome analysis [105]. Another method of BDNF delivery to the retina is intranasally [106,107]. Semmer et al. showed that after intranasal administration, proteins like BDNF reach the retina and optic nerve and increase the expression of pro-survival pathways by increasing the expression of molecules like Phosphorylated Signal Transducer and Activator of Transcription-3 (pSTAT3), the active form of Signal Transducer and Activator of Transcription-3 (STAT3), a transcription factor that regulates the genes responsible for normal cell growth and their differentiation, after an ischemic and traumatic optic neuropathy [108,109]. However, the most practical, safe, and efficient techniques for BDNF delivery to the posterior eye segment must still be discovered so that the target population can benefit from it (Table 2).

MicroRNA (miRNAs) are known as major regulators of gene expression and are involved in the progression of various diseases, including T2D. Certain miRNA clusters are associated with diabetes and exhibit dysregulated expression and activity, prompting interest in their therapeutic use as clinical targets [110]. Lixia Lu et al. showed that BDNF is a suitable target for mir365 mRNA in the diabetic retina, suggesting that mir365 mRNA has a regulatory effect on BDNF. In their study, this interaction was detected in rat Müller cells, with mir-365 levels increasing during the first one to two weeks of diabetes, while BDNF expression significantly decreased after 4 weeks [111]. Similarly, Zhao et al. observed BDNF downregulation by miR-365 in the renal tissue of a diabetic nephropathy (DN) rat model and high glucose-induced human proximal tubule cell line (HK-2 cells), predicting that miR-365 has potential binding sites for BDNF [112].

### 4.2. Physical Exercise

Exercise is known to contribute to BDNF mRNA and protein levels in the serum, retina, and skeletal muscle [113,114,115].

Lawson et al. demonstrated that aerobic exercise, particularly treadmill exercise, has a protective effect on neuronal retina following exposure to toxic bright light. This protection is achieved by increasing retinal BDNF protein levels by 20% compared to inactive mice [116]. However, when mice were treated with a BDNF TrkB receptor antagonist, the benefits of exercise were diminished. However, the minimal amount of exercise needed to promote the BDNF response for protective effects in the retina is yet to be determined [115,116].

He et al. demonstrated that treadmill exercise also promotes RGC survival at 7 days post-axotomy. Additionally, treadmill training increased BDNF levels in the retinas of axotomized eyes by 1.7 times at 7 days and 1.6 times at 14 days post-axotomy compared to inactive rats [113].

### 4.3. Nutritional Interventions

Beta-hydroxybutyrate (BHB), a ketone body molecule, is believed to reduce blood glucose levels in T2D patients and exert a protective role in DR by activating the hydroxycarboxylic acid receptor 2 (HCA2). This activation leads to a reduction in NOD-like receptor protein 3 (NLRP3) inflammasome activity, helping to preserve the retina from DR [117].

Trotta et al. proved that the exogenous administration of BHB in mice with DR increases retinal BDNF levels and reduces abnormal retinal autophagy [118].

In DR, both the marker of autophagosome formation (LC3) and autophagosome–lysosome formation (ATG14) were found to be elevated. These markers are associated with the degradation of Connexin 43 (Cnx43), a gap junction protein that is reduced in DR and linked to retinal vascular cell death, serving as a marker of retinal damage in DR. Trotta et al. further confirmed that BHB reduces autophagy by decreasing levels of BLC3 and ATG14, thereby promoting Cnx43 expression through the BDNF-LC3-ATG14 pathway [118].

In addition to abnormal general autophagy, dysregulated microglial autophagy can impair neuronal functions by affecting lipid peroxidation [119]. BHB has been found to regulate the activation of M1 phenotype microglia, which could be beneficial in DR management [118].

The neurotrophic role of BDNF can be enhanced by flavonoids, a polyphenol sub-group. Flavanones, a type of flavonoid, primarily found in citrus fruits like oranges, bergamots, lemons, and grapefruit [120], particularly Naringenin, exhibit antioxidant properties in streptozotocin (STZ)-induced T1DM rats [121]. This leads to increased levels of BDNF, TrkB, and synaptophysin, helping to prevent neurodegeneration [121]. Another group of flavonoids, flavonols, can be found in numerous vegetables, fruits, tea, and wine. Rutin, a representative of this group, has antiapoptotic effects by decreasing caspase-3 and increasing the expression of both BDNF and NGF in the retina of T1DM rats [122].

Oral Crocin treatment was also proposed as a therapeutic supplement for retinal diseases. Crocin treatment was demonstrated to restore and enhance visual acuity (VA) and visual contrast sensitivity function (VCSF) in light-evoked damaged retinas by activating the BDNF–TrkB pathway [123] (Table 3).

In summary, research indicates that dietary modifications, particularly involving specific polyphenols and their derivatives, can elevate circulating BDNF levels. However, the effects of dietary components remain inconsistent. It is important to acknowledge that studying the link between diet and BDNF levels is challenging due to variations in the types of samples used for BDNF measurement and the specific BDNF variants analyzed. Consequently, further research is necessary to develop nutritional strategies that effectively enhance BDNF levels, with a focus on understanding the interplay between peripheral and central mechanisms.

### 4.4. BDNF as a Standalone or Complementary Treatment Option

One of the major limitations of current anti-VEGF therapies is their inability to preserve neuronal integrity [127]. Anti-VEGF injections effectively reduce vascular leakage and neovascularization, but they do not counteract the progressive neuronal apoptosis that precedes microvascular damage in DR [127]. In contrast, BDNF promotes neuronal survival, synaptic plasticity, and neurovascular interactions through the activation of TrkB receptors [18,19]. By engaging intracellular pathways such as MAPK/ERK and PI3K/Akt, BDNF actively prevents apoptosis and enhances synaptic function, making it a superior candidate for addressing early neurodegeneration in DR [18,19].

In addition, anti-VEGF therapy, primarily administered during the advanced stages of DR when neovascularization and macular edema are already present, and BDNF-based treatment may offer the potential for early-stage intervention [127]. BDNF levels are significantly reduced in the serum and aqueous humor of diabetic patients even before the onset of clinically detectable DR, suggesting the pathophysiological involvement of its deficiency in disease progression and that replenishing BDNF levels through exogenous administration could serve as a preventive strategy rather than merely a symptomatic treatment [42,43,44].

Standard available treatments primarily focus on reducing pathological angiogenesis. However, VEGF inhibition can sometimes lead to adverse effects, including compromised physiological angiogenesis, leading to ischemia and worsening retinal damage [127]. BDNF, on the other hand, not only exerts protective effects on retinal neurons but also helps maintain vascular stability by regulating endothelial tight junction proteins such as ZO-1 and occludin [84]. This dual action makes BDNF a compelling complement to existing treatments.

A significant drawback of BDNF therapy is its delivery and bioavailability. Intravitreal injections of BDNF, while effective in animal models, show limited long-term benefits due to rapid degradation [101]. Anti-VEGF therapies, on the other hand, have well-established dosing regimens with longer-lasting effects [127]. To overcome this challenge, innovative strategies as presented above are being explored for BDNF delivery [10]. Gene therapy approaches have demonstrated prolonged BDNF expression in retinal cells, offering a promising alternative to frequent intravitreal injections [10]. Given the distinct but complementary roles of BDNF and anti-VEGF therapies, a combinatorial approach could maximize therapeutic benefits. While anti-VEGF therapy mitigates vascular complications, the co-administration of BDNF could simultaneously preserve neuronal function and synaptic integrity.

Additionally, combining BDNF with other neurotrophic factors, such as glial cell line-derived neurotrophic factor (GDNF), has shown synergistic effects in enhancing retinal neuron survival, potentially outperforming single-agent therapies [128]. As research advances, integrating BDNF-based treatments with current DR management strategies could significantly enhance patient outcomes, preserving vision and preventing disease progression more effectively than conventional treatments alone.

## 5. Conclusions

As diabetes represents an important socioeconomic burden and is associated with impaired quality of life, there are multiple efforts to discover different remedies and ways to prevent its complications, such as DR [1]. Characterized by progressive damage to the retinal microvasculature, DR encompasses a spectrum of complications, including vascular leakage, capillary occlusion, and neurodegeneration [4]. With the prevalence of diabetes projected to rise, the incidence of DR is expected to escalate, underscoring the urgent need for innovative therapeutic strategies [7].

While current treatment strategies focus primarily on managing advanced vascular complications, they often fail to address the early neurodegenerative processes that play a critical role in disease progression [7]. This gap in therapeutic approaches underscores the urgent need for novel interventions that target both vascular and neuronal pathways.

BDNF, a key player in neuronal growth and survival, has emerged as a promising therapeutic target due to its neuroprotective and neuroregenerative properties, useful in protecting retinal cells and the optic nerve from injuries caused by hypoxia and glucose deprivation through different pathways [8,9]. In type 2 diabetes mellitus, the insufficient amount of BDNF can lead to neuro-retinal apoptosis and degeneration; thus, this review suggests that the exogenous administration of BDNF in DR could be taken into consideration [8,9].

However, due to the complex pathogenesis of DR, the most efficient and safe techniques for BDNF delivery to the posterior eye segment are yet to be discovered, as there is room for further research on its potential in DR treatment.

Exploring BDNF as a therapeutic target not only offers hope for slowing or halting DR progression but also contributes to a broader understanding of the intricate connections between metabolic, vascular, and neuronal health.

By advancing our knowledge of BDNF’s role in DR, we can move closer to developing comprehensive strategies that address the unmet needs in the management of this debilitating condition.

## Figures and Tables

**Table 1:** Main effects of BDNF in DR.

Source	BDNF Effects in Diabetic Retinopathy	
Feng et al. [29]	Neuroprotection	- Prevents RGC and photoreceptor apoptosis
		- Neuronal survival and synaptic plasticity
Afarid et al. [10]	Anti-Inflammatory Effects	- Reduces pro-inflammatory cytokines (e.g., TNF-α, IL-1β, IL-6)
Wu et al. [86]		- Suppresses microglial activation
Reddy et al. [84]	Vascular Stability and Anti-Angiogenic Effects	- Stabilizes the BRB by regulating endothelial tight junction proteins
		- Counteracts VEGF-mediated neovascularization
Reddy et al. [84]	Regulation of Oxidative Stress	- Antioxidant defense mechanisms by increasing the activity of SOD and reducing ROS
		- Mitigates mitochondrial dysfunction
Kimura et al. [27]	Retinal Cell Metabolism	- Activates TrkB signaling
		- Supports mitochondrial bioenergetics under hyperglycemic conditions

**Table 2:** Techniques of BDNF administration.

Source	Type of Administration	Merged Molecule	Outcomes
Khalin I et al. [101]	Intravitreal injections	No merged molecule	Short-term result in RGC survival.
		S-PBN	Improved neuronal survival up to 68%.
		L-NAME	Improved neuronal survival up to 55%.
		Combination of GDNF and BDNF	Higher RGC protection, enhanced RGC survival.
Q-L Fu et al. [104]		Hyaluronic acid, leucine-rich, LINGO-1 antagonist	Long-term protection of RGCs.
Afarid et al. [10]	Gene delivery and gene therapy	Viral vector	Preventing vascular and neuronal degeneration in the early stages of DR.
Semmer et al. [108] Marginean et al. [109]	Intranasal way	No merged molecule	Increased expression of pro-survival pathways after an ischemic and traumatic optic neuropathy.

**Table 3:** The effects of different nutritional compounds on BDNF.

**Source**	**Plant**	**Effect on BDNF Expression**
Wu et al. [123]	Crocin	Activates the BDNF–TrkB pathway.
Matos et al. [121]	Flavanones	Increases levels of BDNF, TrkB, and synaptophysin due to its antioxidant properties.
Ola et al. [122]	Rutin	Increases the expression of both BDNF and NGF in the retina by decreasing caspase-3 levels.
Choi et al. [124]	*Eriobotrya japonica* Lindley	Induces direct modulation of the CREB signaling pathways and increased BDNF concentrations.
Osali A. [125]	Curcumin	Stimulates the PI3K-Akt and CREB pathways, in patients with metabolic syndrome.
Fukuchi et al. [126]	Ginseng Radix	Regulates BDNF transcription via Ca^2+^ signal-mediated CREB-dependent transcription.
